# Preschool Teachers’ Cognitions, Emotions, and Tolerance toward Children’s Hypothetical Social Behaviors in the Classroom

**DOI:** 10.3390/ejihpe14010002

**Published:** 2023-12-20

**Authors:** Maryse Guedes, Manuela Veríssimo, António J. Santos

**Affiliations:** William James Center for Research, ISPA—Instituto Universitário, 1149-041 Lisbon, Portugal; mveriss@ispa.pt (M.V.); asantos@ispa.pt (A.J.S.)

**Keywords:** preschool teachers, cognitions, emotions, tolerance, social behaviors

## Abstract

Teachers’ tolerance toward children’s social behaviors is, in part, guided by teachers’ cognitions and emotions. Few studies have examined the associations between teachers’ cognitions, emotions, and tolerance toward children’s social behaviors. This study aimed to (1) describe the cognitions, emotions, and tolerance of Portuguese preschool teachers toward children’s shy, physically and relationally aggressive, rough-and-tumble play, exuberant, and unsociable behaviors at preschool, depending on children’s sex; and (2) examine the direct and indirect associations (via teachers’ emotions) between teachers’ cognitions and tolerance toward children’s social behaviors, depending on children’s sex. One hundred and seven preschool teachers completed the Child Behaviors Vignettes. Preschool teachers displayed more negative views toward children’s physical and relational aggression, reported positive perspectives toward children’s rough play and mixed attitudes toward children’s exuberance, and differentiated shy from unsociable behaviors. Direct associations between teachers’ cognitions and tolerance were found only for physical aggression. Teachers’ anticipation of negative peer costs and academic performance appear to exert an indirect influence on teachers’ tolerance toward physical aggression and unsociability, via increased levels of worry. These findings highlight the role of teachers’ emotions for tolerance toward children’s social behaviors and the need to enhance their self-awareness.

## 1. Introduction

The preschool classroom is a major developmental context [1]. According to a bioecological developmental framework [1,2], children’s individual characteristics, namely children’s challenging social behaviors in the preschool classroom, are one of the factors that may shape the proximal processes of bidirectional interactions with peers. This theoretical framework [2] establishes that peer interactions in the classroom may be influenced by teachers’ behavioral responses to children’s social behaviors. Within the broader multi-leveled context of internal (e.g., teachers’ years of experience) and external factors (e.g., work setting) [2,3], teachers’ behavioral responses are, in part, enacted by teachers’ beliefs toward children’s challenging social behaviors. Teachers’ beliefs refer to a set of integrated and dynamic views that encompass cognitions [4] that can be used to frame specific problems in the classroom [3], such as causal attributions or anticipated costs of children’s challenging social behaviors [5,6]. Furthermore, teachers’ beliefs encompass an affective component [4], including emotional reactions [1], that may influence the enactment of teachers’ cognitions within behaviors [3]. Exploring teachers’ cognitions, emotional reactions, and behavioral responses to children’s social behaviors is essential to design new teacher-led interventions that can enhance positive peer interactions in the classroom.

### 1.1. Preschool Teachers’ Cognitions, Emotions, and Tolerance toward Children’s Challenging Social Behaviors

To date, many studies have examined the cognitions and tolerance (i.e., likelihood to intervene or not) of North American and Chinese elementary school teachers (from kindergarten to eighth grade) toward children’s challenging social behaviors using a hypothetical vignette method [7]. Globally, extant research found that teachers displayed the least favorable cognitions and behavioral responses toward physical aggression [5,8,9], that is, children’s intent to harm others using physical force [10]. More specifically, teachers were more likely to attribute physical aggression to situational factors but anticipated that this type of behavior would have heightened academic and social costs and would be less tolerated than other types of social behaviors [5,8,9]. A few studies have suggested that teachers appear to distinguish physical aggression from relational aggression [11,12]. Relational aggression, which refers to children’s intent to harm others using efforts to damage relationships [10], was less likely to elicit negative views and was associated with higher levels of tolerance than physical aggression [11,12]. Rough-and-tumble play, described as fighting behaviors with a social dimension [13,14], appeared to be often perceived as aggression and to be less tolerated than other forms of play in the classroom [14]. The attitudes of elementary school teachers toward exhuberance, characterized by high levels of positive affectivity and extroversion that coexist with increased impulsivity [15], were mixed. Participants were more likely to attribute exuberant behaviors to internal factors and to intervene to interrupt them when compared with socially withdrawn behaviors [6,16]. According to teachers, socially withdrawn behaviors, which refer to children’s self-imposed isolation from the peer group [17], were associated with less favorable academic and peer outcomes than exuberant behaviors [6,16,18]. Among socially withdrawn behaviors, teachers distinguished between shyness (i.e., increased wariness and self-consciousness in social situations [17]) and unsociality (i.e., preference for solitude due to a low desire for social engagement [17,19]), in terms of their motivational substrates and consequences. Given their increased social and academic costs, shy behaviors were considered less controllable, less attributable to personality factors, and less tolerable than unsociable behaviors [5].

Despite their contribution, studies conducted in samples of elementary school teachers have not explored the affective component of teachers’ beliefs [4] (namely, emotional reactions) and may not be directly replicated in preschool samples [1]. The few studies conducted in samples of North American and Chinese preschool teachers replicated prior findings, except concerning cognitions toward rough-and-tumble play [1,9,20]. Preschool teachers displayed mixed attitudes toward rough play. Rough play appeared to be more tolerated than aggressive behaviors but was more likely to elicit teachers’ intervention than exuberant and socially withdrawn behaviors [1]. However, ref. [1] found that teachers considered that this type of social behavior would have more positive outcomes in the peer group than the remaining social behaviors. Part of these studies explored the affective component of teachers’ beliefs. Preschool teachers reported higher levels of anger toward agression when compared with social withdrawal [1,20]. However, participants displayed comparable levels of worry about physically aggressive and shy behaviors and these levels were higher than those reported for other social behaviors [1,20]. To the best of our knowledge, little is known about the cognitions, emotions, and behavioral responses of preschool teachers toward children’s challenging social behaviors in European countries, such as Portugal. The balance between collectivist (good manners, responsibility, tolerance, and respect) and individualistic (independence and determination) values [21] that characterize present-day Portuguese society may influence how teachers think, feel, and respond to children’s social behaviors [2,22].

### 1.2. Associations between Teachers’ Cognitions, Emotions, and Tolerance toward Children’s Social Behaviors

Few studies have gone beyond the description of teachers’ perspectives and examined the relationship between teachers’ cognitions, emotions, and behavioral responses to children’s challenging social behaviors. Ref. [9] found that teachers who perceived shy and unsociable behaviors as globally more acceptable were less likely to be tolerant toward them. These associations were not observed for relationally aggressive behaviors [9]. Although relevant for the state of the art, this study examined overall perceptions of acceptability rather than specific cognitions (such as causal attributions and anticipated costs) and did not consider the affective component of teachers’ beliefs. Research has found that teachers’ attributions of greater intentionality are associated with increased anger [23,24], which, in turn, may be more prone to evoke immediate behavioral responses [1]. This may be particularly true for externalizing behaviors that have been found to be associated with teachers’ anger [25,26]. In contrast, teachers’ anticipation of negative costs for children’s developmental outcomes may be associated with more future-oriented emotions, such as worry [1,20]. This type of emotion may, in turn, lead to lower tolerance toward children’s social behaviors, namely socially withdrawn behaviors [1]. However, the indirect associations (via emotions) between teachers’ specific cognitions and tolerance toward children’s challenging social behaviors in preschool classrooms need to be tested.

### 1.3. Teachers’ Beliefs, Emotions and Tolerance, Depending on Teachers’ Sex

Examination of the direct associations and indirect associations (via emotions) between teachers’ cognitions and tolerance toward children’s distinct social behaviors needs to consider the effects of children’s sex. In fact, extant research has identified differences in teachers’ cognitions, emotions, and tolerance toward children’s social behaviors, depending on children’s sex. Globally, teachers considered that boys are less in control of all challenging social behaviors [5] and would display lower academic performance than girls when displaying physical aggression [20]. Inverse sex differences were found concerning anticipated negative peer costs of all challenging social behaviors and concerning anger toward relational aggression [20]. Furthermore, teachers reported higher levels of tolerance toward children’s challenging social behaviors among girls than boys, except shyness [1,5,6,20].

### 1.4. The Present Study

To overcome identified gaps in the literature, this study aimed to: (1) describe the cognitions, emotions, and tolerance of Portuguese preschool teachers toward children’s shy, physically and relationally aggressive, rough play, exhuberant, and unsociable behaviors at preschool, considering the effect of children’s sex; and (2) examine the direct and indirect associations (via teachers’ worry or anger) between teachers’ cognitions and tolerance toward children’s social behaviors, considering the effect of children’s sex.

With respect to the first aim, we hypothetize that preschool teachers would report less favorable attributions of intentionality and anticipated costs, more negative emotions, and lower tolerance toward physical aggression when compared with other social behaviors (H1). The anticipated costs and the tolerance of preschool teachers toward rough play are expected to be mixed (H2). Preschool teachers are expected to report lower intentionality, but more negative anticipated costs and lower levels of tolerance toward shy than unsociable peers (H3). We hypothetize that preschool teachers will report lower intentionality, more anticipated academic costs, less anticipated peer costs, and lower tolerance toward all social behaviors for boys than for girls. The only exception will be tolerance toward shyness (H4).

With respect to the second aim, we expect that causal attributions (increased intentionality) will be associated both directly and indirectly (via higher levels of anger) with lower tolerance toward children’s social behaviors (H5). We also expect that increased future-oriented cognitions (anticipated costs) will be associated both directly and indirectly (via higher levels of worry) with lower tolerance toward children’s challenging behaviors (H6).

## 2. Materials and Methods

### 2.1. Participants

One hundred and seven Portuguese preschool teachers working with children aged 3 to 6 years participated in the study. Teachers were aged, on average, 42 years (*SD* = 9.96). Most teachers held a bachelor’s degree (*n* = 58, 54%), 38% a MSc degree (*n* = 41), 9% (*n* = 1) a PhD degree, and 9% (*n* = 1) a post-graduate qualification. Most teachers were women (*n* = 101, 94%) and had been working as preschool teachers, on average, for 17 years (*SD* = 10.34).

### 2.2. Procedure

The ISPAEthics Committee approved the present study. From December 2021 to June 2022, preschools’ and professional associations’ boards were contacted with the aim of presenting the study aims and procedures.

Preschools’ and professional associations’ boards sent the information provided by the research team (i.e., study aims, procedures, voluntary nature of the participation, confidentiality of the responses, link to the online informed consent form and self-report questionnaires, contact details of the research team), by e-mail, to preschool teachers. Furthermore, preschool teachers from the contact network of the research team and registered with the online professional network LinkedIn were sent the previously described information.

Preschool teachers completed the informed consent before accessing the self-report questionnaires via an online protected platform (Qualtrics). Preschool teachers received the scenarios of the *Child Behaviors Vignettes* in a random order and were randomly assigned vignettes presenting the behaviors of a male (*n* = 52) or female (*n* = 55) preschooler.

### 2.3. Instruments

*Sociodemographic form*: This form collected information on teachers’ age, education, years of professional experience, and work setting (i.e., private, or public preschools).

*Child Behaviors Vignettes* [1,5,20]: This self-report questionnaire consists of six short scenarios, presenting children displaying different social behaviors toward peers in the preschool classroom: (1) shyness (i.e., the target child appears somewhat anxious, inches closer to the other children, but does not try to join in); (2) unsociability (i.e., the target child is playing quietly away from other children, does not appear anxious and, if left undisturbed, would seem likely to happily continue to play on his own); (3) relational aggression (i.e., the target child says to a peer: “if you don’t let me have the purple marker I won’t invite you to my birthday party”); (4) physical aggression (i.e., the target child approaches a peer, grabs his toy, and pushes him down); (5) rough play (i.e., during a game, the target child chases and jumps on another peer’s back and both children fall on the ground laughing); and (6) exuberance (i.e., during circle time, the target child blurts out, frequently interrupts other children, and tends to speak too loudly). Furthermore, the instrument includes two short scenarios representing empathy (i.e., the target child goes to a crying peer, helps him to sit up, and sits together with the other child until she stops crying) and sociability (i.e., the target child invites two peers who are nearby to join a game) toward peers, which were also presented to the preschool teachers for control purposes but were not considered for data analysis. After each scenario, the preschool teachers answered 11 questions to assess causal attributions, anticipated costs, emotions, and tolerance toward children’s presented social behaviors.

To assess behavioral reactions, teachers were asked to rate how tolerant and encouraging they would be (from 1—*Not at All* to 5—*Very*) and the likelihood of not intervening, intervening to stop, and praising (from 1—*Not at All* to 5—*Very Strongly*) each of the presented behaviors. With respect to emotions, teachers were asked to report how strongly they would feel anger, worry, and happiness toward each of the presented behaviors, using a 5-point Likert scale (1—*Not at All* to 5—*Very*). The positive behavioral (i.e., degree of encouragement and likelihood to praise) and emotional (i.e., happy) reactions were included for control purposes but were not considered for data analysis. To assess attitudes and beliefs, teachers were asked to assess the likelihood of each hypothetical child to perform well academically and to be disliked, excluded, and ignored by other children, using a 5-point Likert scale (from 1—*Not at All Likely* to 5—*Very Likely*). Teachers’ attributions of causality, intentionality and stability were also assessed. For each target child, teachers were asked to rate whether the target child acted this way because it was in its nature and personality or whether the behavior might be due to something in that particular classroom (from 1—*Completely due to Personality* to 5—*Completely due to Situation*), acted this way on purpose or might have not meant to act this way (from 1—*Definitively Did it on Purpose* to 5—*Definitely Did Not Mean to do This*), and might be going through a stage that will end soon or might keep on acting this way (from 1—*Definitely a Stage that Will Pass* to 5—*Definitively Will Act this Way in Future*). The latter item was reverse coded.

The *Child Behavior Vignettes* yielded two aggregate scores for teachers’ tolerance (i.e., likelihood of intervening to stop the behavior [reverse coded], likelihood of not intervening, and tolerance) and anticipation of negative peer responses (i.e., likelihood of being disliked, ignored, and excluded by peers) for children’s potentially negative social behaviors [20]. Higher scores in Tolerance mean that teachers are less likely to intervene to interrupt the target behavior. Higher scores in Anticipated Negative Peer Responses mean that teachers consider that the target child is more likely to be disliked, ignored, and excluded by peers. The remaining items (i.e., both negative emotions, anticipated academic performance, and attributions [with stability attributions reverse coded]) relating to children’s potentially negative behaviors were not combined in aggregated scores [5,20]. Higher scores in Worry and Anger mean that teachers are more likely to report such emotions about the target child. Higher scores in Anticipated Academic Performance mean that teachers anticipate that the target child would attain better academic performance in their classroom. Higher scores in Causality, Intentionality, and Stability Attributions indicate that teachers considered the target behavior as more situational, unintentional, and transitory.

Prior research conducted in Canada and China has established the reliability and validity of this measure [1,5,6,20]. Back–forward translation procedures were used to constitute the Portuguese version of the instrument. In the present sample, Cronbach’s alphas for the two aggregate scores were 0.65 (Tolerance) and 0.88 (Negative Anticipated Peer Outcomes).

### 2.4. Data Analysis

With respect to the first aim, preliminary correlation analyses were performed to identify potential sociodemographic covariates. Mixed ANOVAs (for teacher tolerance, negative peer responses, and academic performance) and MANOVAs (for attributions and negative emotions) were performed, using the child’s sex in the presented scenarios as a between-subject factor and the type of social behavior as a within-subject factor. When Type of Behavior or Type of Behavior × Child’s Sex effects were identified, paired *t*-tests with Bonferroni corrections were conducted.

Concerning the second aim, preliminary correlation analyses were performed to identify significant associations between teachers’ cognitions (i.e., anticipated negative peer responses, academic performance, and attributions), emotions (i.e., anger and worry), and tolerance toward children’s social behaviors. When significant associations between the aforementioned teachers’ cognitions, emotions, and tolerance toward one of the assessed children’s social behaviors were identified, simple mediated regression analyses, using the PROCESS macro (model 4) [27], were performed. These simple mediation analyses allowed us to test the indirect effects of teachers’ cognitions on teachers’ tolerance through teachers’ emotions about children’s different social behaviors. When a significant indirect effect was identified, moderated mediation analyses, using the PROCESS macro (model 59) [27] were performed to examine whether the direct and indirect effects of teachers’ cognitions on tolerance, via emotions, varied according to children’s sex. Children’s sex was hypothetized to affect the paths linking teachers’cognitions and emotions, teachers’ emotions and tolerance, and teachers’ cognitions and tolerance. Continuous variables involved in interactions were centered before performing the moderated mediation analyses [27]. Data analyses, using the PROCESS macro, relied on nonparametric bootstrapping, which is considered a valid method to test mediation models and is appropriate for small samples. Post hoc a posteriori power analyses, using G-Power [28,29], showed that medium to large effects could be detected for both study aims.

## 3. Results

### 3.1. Preschool Teachers’ Cognitions, Emotions, and Tolerance toward Children’s Social Behaviors, Considering the Effect of Children’s Sex

Preliminary correlation analyses did not identify covariates for teacher-rated negative peer impact and attributions about the situational and transitory nature of the presented behaviors. However, teachers’ age was positively associated with teacher-rated anticipated academic performance (*r* = 0.21, *p* < 0.001) and tolerance toward relationally aggressive behaviors (*r* = 0.26, *p* < 0.001). Teachers’ years of professional experience were positively associated with attributions of unintentionality to unsociability (*r* = 0.21, *p* < 0.001), but negatively associated with attributions of unintentionality to exuberance (*r* = −0.27, *p* < 0.001) and with worry to rough play (*r* = −0.24, *p* < 0.001).

#### 3.1.1. Teachers’ Cognitions

##### Anticipated Negative Peer Responses

As found in Table 1, a significant main effect of Type of Behavior was identified. Teachers perceived that physically aggressive behaviors would have an increased negative impact in the peer group than shy (*t* = 7.93, *p* < 0.001, *d* = 0.48), rough play (*t* = 13.07, *p* < 0.001, *d* = 1.26), exuberant (*t* = 7.36, *p* < 0.001, *d* = 0.90), and unsociable (*t* = 5.88, *p* < 0.001, *d* = 0.57) behaviors. Similarly, teachers perceived that relationally aggressive behaviors would have an increased negative impact within the peer group when compared with shy (*t* = 2.81, *p* = 0.006, *d* = 0.31), rough play (*t* = 10.66, *p* < 0.001, *d* = 1.07), exuberant (*t* = 5.25, *p* < 0.001, *d* = 0.51), and unsociable (*t* = 3.96, *p* < 0.001, *d* = 0.38) behaviors. However, teachers considered that shy (*t* = 7.93, *p* < 0.001, *d* = 0.76), unsociable (*t* = 6,72, *p* < 0.001, *d* = 0.65), and exuberant (*t* = 7.97, *p* < 0.001, *d* = 0.77) behaviors would have more negative costs in the peer group than rough play.

##### Anticipated Academic Performance

Controlling for teachers’ age, a main significant effect of Type of Behavior was identified (see Table 1). Teachers perceived that hypothetical children displaying rough play would be more likely to have a good academic performance than those exhibiting shy (*t* = 3.58, *p* < 0.001, *d* = 0.35), physically aggressive (*t* = 5.54, *p* < 0.001, *d* = 0.54), relationally aggressive (*t* = 3.87, *p* < 0.001, *d* = 0.38), and exuberant (*t* = 3.70, *p* < 0.001, *d* = 0.36) behaviors. Similarly, teachers perceived that hypothetical children displaying unsociable behaviors would be more likely to have a good academic performance than those displaying shy (*t* = 3.60, *p* < 0.001, *d* = 0.35), physically aggressive (*t* = 5.46, *p* < 0.001, *d* = 0.53), relationally aggressive (*t* = 3.36, *p* < 0.001, *d* = 0.33), and exuberant (*t* = 3.49, *p* < 0.001, *d* = 0.34) behaviors.

##### Attributions

Controlling for teachers’ years of professional experience, a main significant multivariate effect of Type of Behavior was observed (see Table 1). Significant differences were observed in teachers’ attributions about the situational (*F* = 3.19, *p* = 0.01, η^2^p = 0.031), uncontrollable (*F* = 14.14, *p* < 0.001, η^2^p = 0.133), and transitory nature (*F* = 2.24, *p* = 0.049, η^2^p = 0.022) of the presented behaviors.

Exuberant behaviors were less attributed to situations than shy (*t* = −4.07, *p* < 0.001, *d* = 0.40), physically aggressive (*t* = −6.81, *p* < 0.001, *d* = 0.66), relationally aggressive (*t* = −4.23, *p* < 0.001, *d* = 0.31), and rough play (*t* = −6.93, *p* < 0.001, *d* = 0.67) behaviors. Similarly, teachers considered that unsociable behaviors were less due to situations compared with shy (*t* = 3.67, *p* < 0.001, *d* = 0.36), physically aggressive (*t* = −5.54, *p* < 0.001, *d* = 0.54), relationally aggressive (*t* = 3.61, *p* < 0.001, *d* = 0.35), and rough play (*t* = −7.08, *p* < 0.001, *d* = 0.68) behaviors. Nevertheless, teachers considered that shy behaviors were less due to situations than rough play (*t* = −4.48, *p* < 0.001, *d* = 0.43).

With respect to attributions about behaviors’ controllability, teachers perceived physically aggressive behaviors as less unintentional than shy (*t* = −8.28, *p* < 0.001, *d* = 0.80) and exuberant (*t* = −4.40, *p* < 0.001, *d* = 0.43) behaviors. Similar differences were found between relational aggression and shyness (*t* = −10.49, *p* < 0.001, *d* = 1.01) or exuberance (*t* = −10.49, *p* < 0.001, *d* = 1.01). Teachers considered that unsociable behaviors were less unintentional than shy (*t* = −7.50, *p* < 0.001, *d* = 0.73) and exuberant (*t* = −3.33, *p* < 0.001, *d* = 0.32) behaviors, but more unintentional than relational aggression (*t* = 4.04, *p* < 0.001, *d* = 0.39). Rough play behaviors were perceived as less unintentional than shyness (*t* = −5.59, *p* < 0.001, *d* = 0.54), but as more unintentional than relational aggression (*t* = 4.87, *p* < 0.001, *d* = 0.47).

Lastly, teachers perceived physically aggressive behaviors as more transitory than rough play (*t* = 4.11, *p* < 0.001, *d* = 0.40), exuberance (*t* = 4.55, *p* < 0.001, *d* = 0.23), and unsociability (*t* = 4.71, *p* < 0.001, *d* = 0.26).

#### 3.1.2. Teachers’ Emotions

As found in Table 1, a significant main multivariate effect of Type of Behavior was identified, controlling for teachers’ years of professional experience. Significant differences were observed in teachers’ anger (*F* = 12.04, *p* < 0.001, η^2^p = 0.107) and worry (*F* = 4.92, *p* < 0.001, η^2^p = 0.046), depending on Type of Behavior.

Specifically, teachers reported higher levels of anger toward physically aggressive behaviors than shy (*t* = 10.26, *p* < 0.001, *d* = 0.99), rough play (*t* = 7.51, *p* < 0.001, *d* = 0.73), and unsociable (*t* = 12.02, *p* < 0.001, *d* = 1.16) behaviors. Similar differences were observed between relational aggression and shyness (*t* = 7.79, *p* < 0.001, *d* = 0.75), rough play (*t* = 6.36, *p* < 0.001, *d* = 0.62), or unsociability (*t* = 9.42, *p* < 0.001, *d* = 0.91). Exuberant behaviors also elicited higher levels of anger than shyness (*t* = 7.70, *p* < 0.001, *d* = 0.75), rough play (*t* = 5.21, *p* < 0.001, *d* = 0.50), and unsociability (*t* = 9.67, *p* < 0.001, *d* = 0.96). 

Furthermore, teachers reported higher levels of worry about shy (*t* = 6.09, *p* < 0.001, *d* = 0.58), physically aggressive (*t* = 7.42, *p* < 0.001, *d* = 0.72), and relationally aggressive (*t* = 6.60, *p* < 0.001, *d* = 0.64) behaviors compared with rough play. Similar differences were found between exuberance and shyness (*t* = 4.29, *p* < 0.001, *d* = 0.42), physical aggression (*t* = 7.02, *p* < 0.001, *d* = 0.68), or relational aggression (*t* = 5.87, *p* < 0.001, *d* = 0.57). Teachers also displayed lower levels of worry about unsociability compared with shyness (*t* = 4.99, *p* < 0.001, *d* = 0.48), physical aggression (*t* = 6.16, *p* < 0.001, *d* = 0.60), and relational aggression (*t* = 5.08, *p* < 0.001, *d* = 0.53).

#### 3.1.3. Teachers’ Tolerance

As found in Table 1, a significant main effect of Type of Behavior was identified, controlling for teachers’ age. Teachers displayed lower levels of tolerance toward physically aggressive behaviors than shy (*t* = −16.43, *p* < 0.001, *d* = 1.58), rough play (*t* = −14.19, *p* < 0.001, *d* = 1.58), and unsociable (*t* = −14.02, *p* < 0.001, *d* = 1.35) peer behaviors. Similar differences were observed between relationally aggressive behaviors and shyness (*t* = −13.78, *p* < 0.001, *d* = 1.33), rough play (*t* = 12.18, *p* < 0.001, *d* = 1.18), or unsociability (*t* = −12.17, *p* < 0.001, *d* = 1.17). Exuberant behaviors were perceived as more tolerable compared with physically aggressive behaviors (*t* = 4.52, *p* < 0.001, *d* = 0.44), but as less tolerable than shy (*t* = −12.44, *p* < 0.001, *d* = 1.20), rough play (*t* = −12.19, *p* < 0.001, *d* = 1.15), and unsociable (*t* = −11.67, *p* < 0.001, *d* = 1.12) behaviors.

A significant effect of Type of Behavior x Sex was also found. As shown in Table 1, teachers reported significantly lower levels of tolerance toward hypothetical girls who displayed unsociable behaviors than toward hypothetical boys (*t* = 2.33, *p* < 0.02, *d* = 0.45).

### 3.2. Direct and Indirect (via Emotions) Associations between Teachers’ Cognitions and Tolerance, Depending on Children’s Sex

#### 3.2.1. Preliminary Analyses

As shown in Table 2, preliminary correlation analyses showed that preschool teachers who anticipated more negative peer consequences and increased academic performance reported increased worry and lower tolerance toward physical aggression, relational aggression, rough play, and unsociability. For physical aggression, greater anticipated academic performance was associated with increased anger and lower tolerance. For rough play, only associations between greater anticipated negative peer consequences, increased worry, and lower tolerance were found.

With respect to attributional cognition, teachers who attributed greater uncontrollability to physical aggression reported lower tolerance. Teachers who attributed greater uncontrollability to shyness reported increased worry and lower tolerance. Teachers who considered that relational aggression is less transitory reported increased and lower tolerance. Teachers who considered that unsociability is less situational reported increased worry and increased tolerance.

For exuberance, increased anticipated negative peer consequences were associated with lower tolerance, but not with teachers’ emotions, so no mediated regression analyses were performed for this type of social behavior among children.

#### 3.2.2. Direct and Indirect Associations (via Anger) between Preschool Teachers’ Cognitions and Tolerance

The results of the regressions using the PROCESS macro (model 4) examining the mediating role of anger in the associations between preschool teachers’ cognitions and tolerance toward children’s physical aggression, relational aggression, and rough play are presented in Table 3.

Table 3 shows that preschool teachers’ lower levels of anger were associated with increased tolerance toward physical aggression. However, no significant indirect effect of anger in the association between teachers’ anticipation of academic performance and tolerance toward physical aggression was identified.

For relational aggression, no direct or indirect effects (via teachers’ anger) were found between teachers’ attributions of transitory nature and tolerance (see Table 3).

For rough play, preschool teachers’ increased anticipation of peer costs was only indirectly associated with tolerance, via increased levels of anger (see Table 4). No moderating effects of children’s sex were identified [index of moderated mediation: −0.10 (*SE* = 0.15), 95% CI: −0.42/0.19].

#### 3.2.3. Direct and Indirect Associations (via Worry) between Preschool Teachers’ Cognitions and Tolerance

The results of the regressions using the PROCESS macro (model 4) examining the mediating role of worry in the association between preschool teachers’ cognitions and tolerance toward children’s physical aggression, relational aggression, shyness, and unsociability are presented in Table 4.

As shown in Table 4, preschool teachers’ increased anticipation of negative peer consequences was directly and indirectly (via increased levels of worry) associated with lower tolerance toward physical aggression. No moderating effect of children’s sex was found [index of moderated mediation: 0.02 (*SE* = 0.06), 95% CI: −0.11/0.14] using the PROCESS macro (model 59). Preschool teachers’ increased anticipation of academic performance was only indirectly associated with increased tolerance toward physical aggression, via lower levels of worry. No moderating effects of children’s sex were found [index of moderated mediation: 0.02 (*SE* = 0.09), 95% CI: −0.16/0.20].

Controlling for teacher age, Table 4 shows that preschool teachers’ increased worry predicted lower tolerance toward relational aggression. No direct or indirect associations (via teachers’ worry) were found between teachers’ future-oriented cognitions (i.e., anticipated negative peer consequences and academic performance) and tolerance.

For shyness, teachers’ increased attributions of uncontrollability predicted lower tolerance, via increased levels of worry (see Table 4). No direct associations between teachers’ attributions of uncontrollability and tolerance were found. No moderating effect of children’s sex was found [index of moderated mediation: −0.06 (*SE* = 0.09), 95% CI: −0.24/0.13].

For unsociability, Table 4 shows that teachers’ future-oriented cognitions (i.e., increased anticipated peer costs and academic performance) predicted lower tolerance toward unsociability, via increased levels of worry. No direct associations between teachers’ future-oriented cognitions (i.e., increased anticipated peer costs and academic performance) and lower tolerance were found. No moderating effects of children’s sex were found in the indirect associations between teachers’ anticipation of peer costs and their tolerance, via increased worry [index of moderated mediation: −0.10 (*SE* = 0.09), 95% CI: −0.27/0.08]. No moderated effects of children’s sex were found in the indirect associations between teachers’ anticipation of academic performance and their tolerance, via increased worry [index of moderated mediation: 0.20 (*SE* = 0.11), 95% CI: −0.03/0.44].

Table 4 also shows that teachers’ increased attributions of situational causes to unsociability predicted higher levels of tolerance, via lower levels of worry. No significant direct associations between teachers’ increased attributions of situational causes to unsociability and tolerance were found. No moderating effects of children’s sex were found in the indirect associations between teachers’ causal attributions and tolerance, via levels of worry [index of moderated mediation: −0.11 (*SE* = 0.17), 95% CI: −0.42/0.24], using the PROCESS macro (model 59).

## 4. Discussion

In this study, we describe the cognitions, emotions, and tolerance of Portuguese preschool teachers toward children’s social behaviors, considering the effects of children’s sex. We also explore the direct and indirect (via teachers’ emotions) associations between teachers’ cognitions and tolerance, considering the effects of children’s sex.

### 4.1. Preschool Teachers’ Cognitions, Emotions, and Tolerance toward Children’s Social Behaviors, Considering Children’s Sex

In line with prior research [1,5,6,8,9,20], our findings partially support our first hypothesis (H1), showing that teachers displayed more negative views toward the controllability and peer consequences of children’s physically and relationally aggressive behaviors. These negative views coexisted with increased anger and lower tolerance toward physically and relationally aggressive behaviors compared with socially withdrawn behaviors. These findings suggest that teachers are conscious of the disruptive effects of children’s aggressive behaviors on classroom functioning and peer interactions [30] and of their underlying motivations to hurt or harm others [10], which have been described in the literature. Nevertheless, our findings diverge from prior research showing that teachers did not distinguish physical aggression from relational aggression [1,11,12,20]. Relational aggression typically assumes a more direct and overt form (e.g., harmful verbal communication) during early childhood than during middle childhood [31]. This may explain the similar concerns, emotions, and behavioral responses reported by preschool teachers regarding children’s physical and relational aggression. In accordance with the theoretical framework of [2], cultural factors may have influenced the way that teachers think about relational aggression [22]. In fact, it is possible that harmful verbal communication collides with the values of good manners, respect for others, and tolerance that continue to play a role in child-rearing in Portugal [21]. However, cross-cultural studies are needed to test this hypothesis.

The obtained results partially support our second hypothesis (H2). Preschool teachers hold mostly positive views of rough play and appear to be aware of its playful nature (i.e., elicited by circumstances) and adaptive functions in the social domain, which have been documented in the literature [13]. Similar to their Canadian counterparts [1], our participants considered that rough play would be associated with more positive peer and academic outcomes, lower levels of anger and worry, and greater tolerance than aggressive behaviors. Contrary to prior research [1], rough play was considered as tolerable as socially withdrawn behaviors and more tolerable than exuberant behaviors. These findings seem to support the idea that preschool teachers are more focused on the potentialities of rough play in the social domain than on their potential to escalate to aggressive behaviors, due to the young age of the children [13]. In accordance with the bioecological developmental framework [2], time may also influence how caregivers think, feel, and react to children’s social behaviors. In the present study, teachers’ perspectives were assessed during the third wave of the COVID-19 pandemic crisis. Although children experienced fewer prophylactic restrictions during this stage of the pandemic crisis [32], research found that teachers observed a decline in communication and social skills among older children (e.g., [33]). Within this context, it is possible that preschool teachers were more tolerant toward playful interactions that can have benefits for peer interactions.

Preschool teachers in our sample appear to be conscious of the temperamental roots and unintentional nature of exuberance that have been described in the literature [9]. In line with prior research [1,25,26,27,28] and with our second hypothesis (H2), participants in our sample hold mixed perspectives toward exuberant behaviors. Preschool teachers considered that exuberant behaviors would be less concerning and more tolerable than aggressive behaviors. However, participants would be more likely to intervene to interrupt them than socially withdrawn behaviors. Due to their high levels of extroversion and impulsivity [15], exuberant children may demand more attention from teachers to regulate the potential disruptive effects of their verbal interruptions (e.g., talking out of turn) in the classroom [1,6]. Nonetheless, preschool teachers were less concerned and anticipated more positive peer and academic outcomes for exuberant children compared with shy children. Convergent with prior research, we found that exuberance may be associated with peer acceptance [34] and teachers’ ratings of better academic performance, due to increased behavioral engagement in the classroom [1,6].

Our findings partially support our third hypothesis (H3). Similar to their Canadian and Chinese counterparts [1,5,20], preschool teachers in our sample were able to identify the distinct motivational and psychological substrates underlying shy and unsociable behaviors [35]. In line with the developmental literature, participants acknowledged that the reduced desire for social engagement associated with unsociability [19] appears to reflect a deliberate and intrinsic non-fearful preference for playing alone [36] and were less concerned with this type of social behavior compared with shyness. Contrary to shy children, the literature establishes that unsociable children do not avoid social interaction when asked to participate by teachers and peers [19]. Due to their reduced participation in the classroom, teachers may evaluate the academic outcomes of shy children negatively [37]. In contrast, unsociable children may be more prone to engage in classroom activities when asked to [19] and their solitary behaviors may be interpreted by teachers as being on task [38].

Notwithstanding these similarities, preschool teachers in our sample did not distinguish between shyness and unsociability in terms of anticipated negative peer outcomes. These findings diverge from the perspectives of Chinese and Canadian teachers [1,6,20] and from prior research, suggesting that unsociability is relatively benign during early childhood [39]. As previously stated, our findings need to be interpreted considering macro-time factors [2]. Given the timing of the data collection, it is possible that preschool teachers were more conscious of the potentially negative peer consequences of socially withdrawn behaviors in the classroom during early childhood, independent of their underlying motivational substrates. 

Contrary to our hypothesis (H4), our findings evidenced only subtle sex differences in teachers’ tolerance toward unsociable behaviors. More specifically, preschool teachers in our sample considered that unsociable behaviors were less tolerable among girls than among boys. Inverse sex differences were found for unsociability in a sample of elementary school teachers [5]. These findings are consistent with the greater prosocial orientation of girls during peer interactions [40]. It is plausible that teachers perceive reduced desire to engage in peer interactions, which is related to unsociable behavior [19], as deviant from the normative expectations for girls. This may be particularly salient in Portuguese society, in which collectivist values related to good manners and respect for others continue to be perceived as desirable qualities for children [21].

No significant sex differences were found in teachers’ cognitions, emotions, and tolerance toward the remaining social behaviors. The relatively few sex differences converge with the idea that teachers’ training and experience may counteract sex stereotypes relating to children’s social behaviors [1,5,6]. Nevertheless, these findings need to be interpreted with caution, since teachers were randomly assigned vignettes depicting either boys or girls in the present study.

### 4.2. Direct and Indirect Associations (via Anger) between Preschool Teachers’ Cognitions and Tolerance toward Children’s Social Behaviors

Contrary to our hypothesis (H5), our findings did not identify direct and indirect associations (via anger) between teachers’ causal attributions (i.e., increased intentionality) and lower tolerance toward all the social behaviors of the children. In contrast, our findings support extant theory that acknowledges the relevance of the affective component of teachers’ beliefs [3,4] for the enactment of teachers’ behavioral responses. In fact, teachers’ anger emerged as the only predictor of lower tolerance toward physical aggression and exuberance. The harmful actions of physically aggressive children and the verbal interruptions of exuberant children have an immediate disruptive impact in the classroom for peers and teachers [1,6] that is more likely to elicit emotions of anger [25,26]. Given the instant reaction evoked by basic primary emotions [41], teachers who experience heightened levels of anger in response to such behaviors are more prone to intervene to interrupt them.

Our findings also diverge from our hypotheses, because increased anger mediated the negative associations between teachers’ future-oriented cognitions (i.e., anticipated negative peer costs) and tolerance toward rough play. Research has found that rough play may be misinterpreted by teachers as aggression [14], which has been found to have negative consequences for peer interactions [30]. This kind of negative view may evoke instant emotional reactions, like anger [1], and, in turn, increase teachers’ proneness to intervene to stop rough play in the classroom.

### 4.3. Direct and Indirect Associations (via Worry) between Preschool Teachers’ Cognitions and Tolerance toward Children’s Social Behaviors

Our findings partially support our sixth hypothesis (H6). Direct positive associations between future-oriented cognitions (i.e., anticipated costs) and tolerance were limited to physical aggression. These findings converge with the idea that the direct relationship between beliefs and practices is not always consistent within the broader multi-leveled context in which teachers interact with preschoolers [2,4]. Children’s overt deliberate actions to hurt or harm others using physical force [10] have been found to be particularly disruptive for peer interactions [30] and may lead to children’s active isolation by peers [17]. Consistent with this idea, research conducted in different countries has consistently found that preschoolers are less prone to display affiliative preferences toward physically aggressive peers compared with socially withdrawn peers (e.g., [42,43,44]). Teachers have also appeared to be conscious of the disruptive nature of physically aggressive behaviors for peer interactions compared with children’s other social behaviors [1,6,20]. Consequently, teachers’ greater awareness concerning the disruptive nature of physically aggressive behaviors for peer interactions may be sufficient to lower their tolerance toward them.

Consistent with our hypothesis (H6), teachers’ future-oriented cognitions (i.e., anticipated costs) appear to exert an indirect influence on teachers’ tolerance, via increased levels of worry, for physical aggression and unsociability. Research has shown that teachers anticipate the most negative future consequences as a result of physical aggression (e.g., [1,5,20]). Notwithstanding their non-fearful preference for solitude [17], unsociable children are able to positively engage with peers or teachers in the classroom [19]. Consequently, teachers may be more likely to think about the long-term consequences of unsociability [1] for children’s developmental outcomes. Teachers who are more likely to think about the future peer and academic experiences of physically aggressive and unsociable children may be more prone to experience complex secondary emotions, like worry [1] and, consequently, to intervene to modify such behaviors.

For shyness, future-oriented cognitions were not directly and indirectly associated (via teachers’ worry) with tolerance. Contrary to the expectation, teachers’ attributions of lower intentionality were associated with lower tolerance, through increased levels of worry. These findings support the idea that socially withdrawn behaviors may be associated with increased worry [1], although not while teachers think about children’s future negative outcomes. Instead, teachers’ attributions regarding the unintentional nature of children’s shy behaviors were significantly associated with teachers’ worry. It is possible that teachers who perceive shy behaviors as less intentional are more conscious of the motivational substrates underlying shyness that have been described in the literature [35], recognizing that shyness reflects conflicting desires for social approach and avoidance, due to social anxiety [17]. Teachers’ concerns about the avoidance–approach conflict underlying shy behaviors [17] may, in turn, reduce their tolerance toward them. In contrast with shy behaviors, teachers’ attributions of external causality were associated with lower tolerance toward unsociable behaviors, through increased levels of worry. It is possible that teachers who perceive unsociable behaviors as more due to situational circumstances are less conscious that these behaviors reflect a preference for solitude [17] and may misinterpret them as shyness, so they are more worried and prone to intervene to modify them.

For relational aggression, teachers’ worry was the only predictor of tolerance, supporting the role of the affective component of teachers’ beliefs in the enactment of teachers’ behaviors [4]. In the short term, relational aggression does not encompass an immediate risk of children’s physical injury and may, thus, be less disruptive to classroom functioning than physical aggression [1]. This type of social behavior may elicit increased future-oriented secondary emotions [44], such as worry. Teachers who experience increased worry are more likely to intervene to interrupt relational aggression in the classroom.

### 4.4. Limitations and Future Directions

This study has limitations. The sample was recruited using a convenience sampling method, and post hoc power analyses showed that medium to large but not small effects could be detected. The measure that was used has been shown to be reliable and valid in different cultures and allowed us to describe teachers’ perspectives of children’s challenging social behaviors. However, this measure only assessed teachers’ perspectives, using hypothetical scenarios and a narrow number of items. It is possible that teachers’ cognitions, emotions, and behaviors are different in real preschool settings. Due to its cross-sectional design, this study did not allow us to establish the direction of the relationships between the variables. The timing of data collection may have also impacted teachers’ perspectives of children’s social behaviors in the preschool classroom.

In future studies, researchers need to combine hypothetical vignette methods with qualitative interviews of teachers or preschool observations and explore both maladaptive and adaptive social behaviors of children over time in different cultures. Differences in teachers’ cognitions, negative emotions, and behavioral responses, depending on children’s sex, need to be explored in more depth, namely regarding socially withdrawn (i.e., unsociable and shy) behaviors. In future studies, the period of the school year in which teachers’ cognitions, emotions, and tolerance are collected also needs to be accounted for, because it can influence the cognitive, affective, and behavioral responses of both teachers and children. Potential bidirectional associations between teachers’ cognitions and tolerance need to be examined. The moderating role of teachers’ individual (e.g., self-efficacy or other ability-related beliefs, personality traits, experience) and contextual (e.g., child-to-teacher ratio, classroom climate) factors in the direct and indirect associations (via a wider range of teachers’ emotions) between teachers’ cognitions and tolerance needs to be examined.

## 5. Conclusions

Globally, our findings show that preschool teachers displayed more negative views toward children’s physically and relationally aggressive behaviors, reported positive perspectives toward children’s rough play, had mixed attitudes toward children’s exuberance, and acknowledged that shyness and unsociability have distinct underlying motivations. Direct associations between teachers’ cognitions and tolerance were only found for physical aggression. Teachers’ anticipation of negative peer costs and academic performance appear to exert an indirect influence on teachers’ tolerance toward physical aggression and unsociability, via increased levels of worry. For shyness, teachers’ attributions of greater uncontrollability were associated with lower tolerance toward shy behaviors, through increased levels of worry.

From an intervention standpoint, our findings suggest that novel teacher-led interventions need to be designed. These intervention programs need to combine the restructuration of dysfunctional interpretations of children’s social behaviors in the classroom with the promotion of teachers’ emotional regulation skills to manage such behaviors. Enhancing teachers’ self-awareness regarding the cognitive and affective components of their belief systems is crucial to empowering them and coaching them in the implementation of empirically validated techniques that can promote children’s positive peer interactions in the classroom.

## Figures and Tables

**Table 1:** Descriptive Statistics (Means and Standard Deviations) of Preschool Teachers’ Attitudes, Beliefs, and Emotional and Behavioral Reactions to Shy, Physically and Relationally Aggressive, Rough Play, Exuberant, and Unsociable Behaviors in the Classroom.

	Shy			Relationally Aggressive			Physically Aggressive			Rough Play			Exuberant			Unsociable		
	Girl	Boy	Total	Girl	Boy	Total	Girl	Boy	Total	Girl	Boy	Total	Girl	Boy	Total	Girl	Boy	Total
	*M* (*SD*)	*M* (*SD*)	*M* (*SD*)	*M* (*SD*)	*M* (*SD*)	*M* (*SD*)	*M* (*SD*)	*M* (*SD*)	*M* (*SD*)	*M* (*SD*)	*M* (*SD*)	*M* (*SD*)	*M* (*SD*)	*M* (*SD*)	*M* (*SD*)	*M* (*SD*)	*M* (*SD*)	*M* (*SD*)
Negative peer responses	2.26 (1.07)	2.40 (0.95)	2.33 (1.01)	2.56 (1.21)	2.76 (0.99)	2.66 (1.11)	2.70 (1.01)	3.07 (1.05)	2.88 (1.04)	1.40 (0.57)	1.71 (0.65)	1.54 (0.63)	2.23 (0.95)	2.25 (0.92)	2.24 (0.94)	2.19 (1.14)	2.27 (0.98)	2.23 (1.06)
Mixed ANOVA statistics, controlling for teachers’ age. Type of Behavior: *F* = 3.47 **, η^2^p = 0.033; Sex: *F* = 0.44, η^2^p = 0.004; Type of Behavior × Sex: *F* = 0.88, η^2^p = 0.009.																		
Academic performance	3.93 (0.92)	3.92 (0.87)	3.92 (0.89)	3.87 (1.02)	3.96 (0.85)	3.92 (0.94)	3.89 (0.92)	3.71 (0.92)	3.80 (0.92)	4.24 (0.81)	4.16 (0.83)	4.20 (0.82)	4.02 (0.89)	3.90 (0.88)	3.96 (0.88)	4.25 (0.82)	4.08 (0.77)	4.17 (0.80)
Mixed ANOVA statistics, controlling for teachers’ age. Type of Behavior: *F* = 41.13 ***, η^2^p = 0.481; Sex: *F* = 1.83, η^2^p = 0.017; Type of Behavior × Sex: *F* = 0.86, η^2^p = 0.008.																		
Situational nature	2.70 (0.57)	2.94 (0.60)	2.81 (0.59)	2.80 (0.74)	2.98 (0.76)	2.88 (0.75)	3.06 (0.66)	2.94 (0.58)	3.00 (0.58)	3.09 (0.73)	3.19 (0.45)	3.14 (0.61)	2.56 (0.65)	2.46 (0.72)	2.51 (0.69)	2.54 (0.69)	2.62 (0.61)	2.58 (0.65)
Unintentionality	3.52 (0.67)	3.54 (0.68)	3.53 (0.67)	2.46 (0.82)	2.58 (0.85)	2.52 (0.83)	2.72 (1.07)	2.85 (0.68)	2.78 (0.88)	3.09 (0.85)	2.85 (0.77)	2.98 (0.82)	3.17 (0.82)	3.29 (0.72)	3.23 (0.70)	2.96 (0.75)	2.85 (0.58)	2.91 (0.68)
Transitory nature	3.31 (0.70)	3.42 (0.61)	3.36 (0.66)	3.31 (0.77)	3.35 (0.70)	3.33 (0.74)	3.56 (0.72)	3.41 (0.65)	3.49 (0.69)	3.13 (0.69)	3.13 (0.61)	3.13 (0.64)	3.16 (0.69)	3.14 (0.68)	3.16 (0.69)	3.13 (0.58)	3.15 (0.46)	3.14 (0.53)
Mixed MANOVA statistics, controlling for teachers’ years of experience. Type of Behavior: Pillai’s trace = 0.49, *F* = 5.50 ***, η^2^p = 0.497; Sex: Pillai’s trace = 0.02, *F* = 0.65, η^2^p = 0.020. Type of Behavior × Sex: Pillai’s trace = 0.17, *F* = 1.18, η^2^p = 0.172.																		
Anger	1.44 (0.63)	1.34 (0.63)	1.39 (0.73)	2.57 (1.33)	2.28 (1.03)	2.43 (1.20)	2.63 (1.14)	2.46 (0.95)	2.55 (1.05)	1.65 (0.85)	1.58 (0.91)	1.62 (0.87)	2.24 (1.09)	2.34 (1.10)	2.29 (1.09)	1.15 (0.53)	1.28 (0.64)	1.21 (0.59)
Worry	3.41 (1.13)	3.56 (1.14)	3.48 (1.13)	3.56 (1.14)	3.50 (1.09)	3.53 (1.14)	3.66 (1.15)	3.61 (1.12)	3.63 (1.13)	2.52 (1.27)	2.52 (1.21)	2.52 (1.24)	2.91 (1.19)	2.88 (1.14)	2.89 (1.16)	2.87 (1.30)	2.84 (1.11)	2.86 (1.21)
Mixed MANOVA statistics, controlling for teachers’ years of experience. Type of Behavior: Pillai’s trace = 0.45, *F* = 7.72 ***, η^2^p = 0.457; Sex: Pillai’s trace = 0.01, *F* = 0.25, η^2^p = 0.005. Type of Behavior × Sex: Pillai’s trace = 0.08, *F* = 0.82, η^2^p = 0.082.																		
Tolerance	3.44 (0.85)	3.50 (0.75)	3.47 (0.80)	2.02 (0.90)	2.08 (0.79)	2.05 (0.85)	1.90 (0.92)	1.79 (0.71)	1.85 (0.82)	3.55 (0.89)	3.46 (0.99)	3.51 (0.94)	2.26 (0.96)	2.10 (0.63)	2.18 (0.82)	3.38 (0.99)	3.81 (0.90)	3.58 (0.96)
Mixed ANOVA statistics, controlling for teachers’ age. Type of Behavior: *F* = 11.75 ***, η^2^p = 0.102; Sex: *F* = 0.07, η^2^p = 0.001. Type of Behavior × Sex: *F* = 2.26 *, η^2^p = 0.021.																		

Note. *M* refers to mean. *SD* refers to standard deviation. *** *p* < 0.001. ** *p* < 0.01. * *p* < 0.05.

**Table 2:** Correlations between Teachers’ Cognitions, Emotions, and Tolerance Toward Children’s Social Behaviors.

	Emotions and Tolerance																	
	Physical Aggression			Relational Aggression			Rough Play			Exuberance			Shyness			Unsociability		
	Ang	Wor	Tol	Ang	Wor	Tol	Ang	Wor	Tol	Ang	Wor	Tol	Ang	Wor	Tol	Ang	Wor	Tol
*Anticipated costs*																		
Academic performance	−0.22 *	−0.29 **	0.23 *	−0.17	−0.27 **	0.22 *	−0.15	−0.12	−0.00	0.01	−0.15	−0.17	0.18	−0.03	0.07	−0.03	−0.26 **	0.17
Peer negative responses	0.16	0.25 **	−0.33 **	0.15	0.25 **	−0.27 **	0.38 **	0.06	−0.14	0.16	0.09	−0.19 *	0.18	0.20	−0.16	0.18	0.35 **	−0.13
*Attributions*																		
Unintentionality	0.17	0.15	−0.24 *	0.16	0.03	0.18	0.03	0.15	−0.08	0.13	−0.00	−0.06	−0.09	−0.28 **	−0.11	0.04	−0.04	0.05
Transitory nature	−0.03	−0.19 *	0.06	−0.20 *	−0.15	−0.31 **	0.16	0.05	−0.23 *	−0.05	−0.02	0.06	−0.07	−0.05	0.16	0.07	0.07	0.09
Situational nature	0.05	0.02	0.11	0.03	−0.04	0.06	0.02	0.09	−0.02	0.04	−0.17	−0.00	−0.02	−0.14	−0.02	0.09	−0.24 *	−0.01
*Tolerance*	−0.26 **	−0.30 **	-	−0.22 *	−0.26 **	-	−0.25 **	−0.48*	-	−0.20 *	−0.12	-	0.01	−0.22 *	-	−0.03	−0.47 **	-

Ang refers to anger. Wor refers to worry. Tol refers to tolerance. * *p* < 0.05. ** *p* < 0.01.

**Table 3:** The mediating role of preschool teachers’ anger in the associations between teachers’ cognitions and tolerance toward children’s social behaviors.

	Tolerance	
	*Β (SE)*	95% CI
Physical aggression		
Ant. acad. perf.	0.16 (0.08)	[−0.01/0.33]
Anger	−0.17 (0.07) *	[−0.32/−0.03] *
Final model	*F* = 5.74, *p* < 0.001, *R*^2^ = 0.10	
Total effect	0.20 (0.08) *	[0.04/0.38]
Direct effect	0.16 (0.09)	[−0.01/0.33]
Indirect effect	0.04 (0.03)	[−0.00/0.11]
Relational aggression		
Teacher age	0.02 (0.01) *	[0.00/0.03] *
Transitory nature	−0.14 (0.11)	[−0.35/0.08]
Anger	−0.12 (0.07)	[−0.26/0.00]
Final model	*F* = 4.70, *p* < 0.001, *R*^2^ = 0.12	
Total effect	−0.18 (0.11)	[−0.40/0.04]
Direct effect	−0.14 (0.11)	[−0.21/−0.35]
Indirect effect	−0.04 (0.03)	[−0.12/0.01]
Rough play		
Ant. peer costs	−0.07 (0.15)	[−0.38/0.23]
Anger	−0.25 (0.11) *	[−0.47/−0.04] *
Final model	*F* = 3.61, *p* = 0.030, *R*^2^ = 0.07	
Total effect	−0.21 (0.15)	[−0.49/0.08]
Direct effect	−0.08 (0.15)	[−0.39/0.23]
Indirect effect	−0.13 (0.07)	[−0.28/0.01]

Ant. acad. perf. refers to anticipated academic performance. Ant. peer costs refers to anticipated negative peer consequences. * *p* < 0.05.

**Table 4:** The mediating role of preschool teachers’ worry in the association between teachers’ cognitions and tolerance toward children’s social behaviors.

	Tolerance	
	*Β (SE)*	95% CI
Physical aggression		
Ant. peer costs	−0.21 (0.07) **	[−0.36/−0.07] **
Worry	−0.17 (0.07) *	[−0.30/−0.03] *
Final model	*F* = 9.95, *p* < 0.001, *R*^2^ = 0.16	
Total effect	−0.36 (0.07) ***	[−0.40/−0.11] ***
Direct effect	−0.21 (0.07) **	[−0.36/−0.07] **
Indirect effect	−0.05 (0.03)	[−0.11/−0.00]
Ant. acad. perf.	0.13 (0.08)	[−0.03/0.31]
Worry	−0.18 (0.07) **	[−0.32/−0.05] **
Final model	*F* = 6.50, *p* < 0.001, *R*^2^ = 0.11	
Total effect	0.20 (0.08) *	[0.04/0.37] *
Direct effect	0.13 (0.09)	[−0.03/0.31]
Indirect effect	0.06 (0.04)	[0.01/0.16]
Relational aggression		
Teacher age	0.01 (0.01) *	[0.00/0.03] *
Ant. peer costs	−0.13 (0.07)	[−0.28/0.01]
Worry	−0.16 (0.07) *	[−0.30/−0.01] *
Final model	*F* = 6.34, *p* < 0.001, *R*^2^ = 0.16	
Total effect	−0.19 (0.07) **	[−0.33/−0.05] **
Direct effect	−0.16 (0.07) *	[−0.30/0.01] *
Indirect effect	−0.03 (0.02)	[−0.08/0.01]
Teacher age	0.02 (0.01) *	[0.00/0.04] *
Ant. acad. perf.	0.10 (0.09)	[−0.07/0.28]
Worry	−0.17 (0.07)	[−0.31/−0.03]
Final model	*F* = 5.72, *p* = 0.001, *R*^2^ = 0.14	
Total effect	−0.19 (0.07) *	[−0.33/−0.05] *
Direct effect	−0.17 (0.07)	[−0.31/−0.02]
Indirect effect	−0.02 (0.02)	[−0.08/0.01]
Shyness		
Unintent.	−0.22 (0.12)	[−0.46/0.00]
Worry	−0.19 (0.07) **	[−0.33/−0.55] **
Final model	*F* = 4.55, *p* = 0.012, *R*^2^ = 0.08	
Total effect	−0.13 (0.12)	[−0.36/0.09]
Direct effect	−0.23 (0.12)	[−0.46/0.05]
Indirect effect	0.09 (0.04)	[0.01/0.19]
Unsociability		
Ant. peer costs	0.04 (0.08)	[−0.12/0.20]
Worry	−0.38 (0.07) ***	[−0.53/−0.24] ***
Final model	*F* = 14.93, *p* < 0.001, *R*^2^ = 0.22	
Total effect	−0.12 (0.08)	[−0.29/0.05]
Direct effect	0.04 (0.08)	[−0.12/0.20]
Indirect effect	−0.15 (0.04)	[−0.25/−0.07]
Ant. acad. perf.	0.17 (0.11)	[−0.03/0.39]
Worry	−0.34 (0.07) ***	[−0.48/−0.20] ***
Final model	*F* = 16.53, *p* < 0.001, *R*^2^ = 0.24	
Total effect	0.31 (0.11) **	[0.09/0.55] **
Direct effect	0.17 (0.11)	[−0.04/0.39]
Indirect effect	0.14 (0.06)	[0.04/0.27]
Sit. nature	−0.19 (0.13)	[−0.45/0.07]
Worry	−0.39 (0.07) ***	[−0.53/0.28] ***
Final model	*F* = 16.14, *p* < 0.001, *R*^2^ = 0.24	
Total effect	−0.01 (0.15)	[−0.30/0.27]
Direct effect	−0.19 (0.14)	[−0.45/0.07]
Indirect effect	0.17 (0.07)	[0.05/0.33]

Ant. peer costs refers to anticipated negative peer consequences. Ant. acad. perf. refers to anticipated academic performance. Unintent. refers to unintentionality. Sit. nature refers to situational nature. * *p* < 0.05. ** *p* < 0.01. *** *p* < 0.001.

## Data Availability

The raw data supporting the conclusions of this article will be made available by the authors upon request.
